# Annotations

**Published:** 1906-04-21

**Authors:** 


### ANNOTATIONS.

### The Marriage of Defectives.

It would not be uncomplimentary to describe the United States as a community in which social experiments are being tried on an extensive scale. Free from the conservative and restraining influence of a large body of inherited tradition, both the individual citizen and the governing authorities are constantly ready to welcome novel proposals and to embark hopefully on policies which elsewhere would need years of hesitation and discussion. Perhaps more particularly does this tendency exhibit itself in connection with legislative suggestions which promise the removal of much individual suffering, or which anticipate a considerable measure of social amelioration. An example of this spirit may be seen in a Bill recently presented to the New York State Legislature for the regulation, or rather for the prohibition, of the marriage of "insane, epileptic, imbecile, and feeble-minded persons." Not only is it proposed that it shall be illegal for any person in one or other of these categories to marry, but also that any such marriage shall be null and void. Whether public opinion will support so marked an invasion of individual liberties remains to be seen, but it can hardly be doubted that the mere introduction of such a suggestion to a responsible legislative body is an indication of an increasing recognition of the right of the State to regulate the conduct of its citizens, even in their private relationships. The fact is that with the growing complexities of civilisation it is more and more plainly realised that the acts of the individual touch and modify the responsibilities of the community, and even the most ardent anti-Socialist will hardly question the right of the State to restrain actions which, by multiplying the unfit, cause an addition to the charges made upon the municipal or imperial exchequer. Whatever other rights may be questioned, the right of the community to protect itself from unfair burdens must surely be recognised.

### Chinese Surgery.

Some interesting statements have been made by a medical man in Hongkong in respect to Chinese surgery. Alluding to a distinction between internal and external medicine, the external medicine being what we call surgery, he referred to the fact that several hundred years before the birth of Christ there was an eminent Chinese surgeon who believed in extensive operations and amputations, but he was almost alone, as no one else ever attempted even to cut off a finger. Describing the chief aims of the native doctor in surgery at the present time, he said that they are the use of the needle and counter irritation, the latter including what is ordinarily termed massage, and burning of the flesh. He had himself seen children treated by this burning process for diseases of the stomach. Commenting on the deplorable ignorance of anatomy among the Chinese, he affirmed that they have an idea that the heart and the stomach are connected, and that the epigastrium is the seat of thought. They also imagine that the gall-bladder is the seat of boldness, and that all schemes originate from it. He attributed the difficulty of introducing the science of Western medicine into China to intense hatred of the foreigner on the part of the native; and in confirmation of this belief he mentioned that of more than a score of graduates of the Hongkong College of Medicine not one had settled in China. He had personally known Chinese communities indulge in a riot over the incoming of a medical man educated in Western science, the disturbance being promoted by native doctors, who could not, therefore, possess any profound faith in the stability of their practice. This, perhaps, is the most hopeful sign of the times in the Celestial Empire, so far as the practice of medicine in any of its branches is concerned.

### The Cerebro-Spinal Fluid in Tabes Dorsalis and General Paralysis.

In his Lumleian lectures on Tabes Dorsalis, Dr. David Ferrier recently discussed the value of the examination of the cerebro-spinal fluid as a means of diagnosis. In health the centrifugalised deposit from the fluid contains scarcely any cellular elements beyond an occasional endothelial cell, and perhaps one to three lymphocytes in a field with a magnification of 400 diameters. As opposed to this, in the acute microbic affections of the cerebro-spinal meninges there is a leucocytosis in which the leucocytes are of the polynuclear type, whilst in chronic syphilitic and tuberculous affections of the meninges there is excess of mononuclear leucocytes (lymphocytes). Further, and this is the point now at issue, both in tabes and in general paralysis, from the earliest to the latest stages, there is also a marked lymphocytosis of the cerebro-spinal fluid. Instead of two or three lymphocytes, as in the normal condition, the field of the microscope may contain great numbers, even several hundreds. Dr. Ferrier has found himself able to rely on this fact in the diagnosis of general paralysis even before there were any abnormalities of the pupils or other physical signs characteristic of the disease. At first sight the fact of lymphocytosis in tabes dorsalis would seem to confirm the views of those who hold that the disease depends on a syphilitic affection of the meninges. But opposed to this is the occurrence of the same phenomenon in other affections, such as Landry's paralysis, subacute combined degeneration, herpes zoster, etc., in which no question of a meningitis arises. And still more conclusive is the observation that the lymphocytosis of tabes and of general paralysis does not yield in the slightest degree to the most energetic anti-syphilitic treatment, whereas, as shown by Purves Stewart, the opposite is the case in definite and active syphilitic disease of the nervous system.

